# Suppression of Nestin reveals a critical role for p38-EGFR pathway in neural progenitor cell proliferation

**DOI:** 10.18632/oncotarget.13498

**Published:** 2016-11-22

**Authors:** Wentao Hu, Hong Lu, Shang Wang, Wenhan Yin, Xujie Liu, Lin Dong, Richard Chiu, Li Shen, Wen-Jing Lu, Feng Lan

**Affiliations:** ^1^ Department of Neurology, The First Affiliated Hospital of Zhengzhou University, Zhengzhou, Henan, China; ^2^ Beijing Institute of Heart, Lung and Blood Vessel Diseases, Beijing, China; ^3^ Beijing Lab for Cardiovascular Precision Medicine, Capital Medical University, Beijing, China; ^4^ The Key Laboratory of Remodeling-Related Cardiovascular Disease, Ministry of Education, Beijing, China; ^5^ Beijing Collaborative Innovation Center for Cardiovascular Disorders, Anzhen Hospital, Capital Medical University, Beijing, China; ^6^ Deparment of Radiological Medicine, Chongqing Medical University, Chongqing, China; ^7^ Department of Cell Biology Peking University Health Science Center, Beijing, China; ^8^ Deparment of Radiology, Stanford University School of Medicine, Stanford, California, USA

**Keywords:** nestin, NPCs, proliferation, EGFR, self-renewal

## Abstract

The expression of intermediate filament Nestin is necessary for the neural progenitor cells (NPCs) to maintain stemness, but the underlying cellular and molecular mechanism remains unclear. In this study, we demonstrated that Nestin is required for the self-renew of NPCs through activating MAPK and EGFR pathways. Knockdown of Nestin by shRNA inhibited cell cycle progression and proliferation in mouse NPCs. Moreover, suppression of Nestin reduced expression of the epidermal growth factor receptor (EGFR) in NPCs and inhibited the mitogenic effects of EGF on these cells. Treatment of NPCs with p38-MAPK inhibitor PD169316 reversed cell cycle arrest caused by the knockdown of Nestin. Our findings indicate that Nestin promotes NPC proliferation via p38-MAPK and EGFR pathways, and reveals the necessity of these pathways in NPCs self-renewal.

## INTRODUCTION

Nestin is a type IV intermediate filament protein expressed primarily in neuroepithelial stem cells and proliferating neural progenitor cells (NPCs) [1, 2]. Upon differentiation, Nestin expression in NPCs is dramatically down-regulated and replaced by other intermediate filament proteins, such as neurofilament in neurons, and glial fibrillary acidic protein (GFAP) in astrocytes and mature neural cells [1]. Because Nestin is expressed predominantly by NPCs during development, the protein has traditionally been recognized as a marker of NPCs.

Recent studies have indicated that Nestin is also expressed by many other progenitor cell types besides NPCs, such as progenitors of cardiac, skeletal and smooth muscle, endothelial, hepatic, and pancreatic tissues [3–8]. It has been postulated that Nestin is a universal stem cell marker shared by all progenitors and stem cells, and plays a vital role in maintaining their progenitor properties. Moreover, Nestin is also expressed in malignant tumor cells and is associated with tumor cell proliferation and metastasis in colorectal [9], breast [10], prostate [11], and pancreatic [12] cancers. Given that proliferation and self-renewal are features shared by progenitor/stem cells and cancer cells, the up-regulation of Nestin in both cell types suggests that Nestin is involved in the proliferation and growth of NPCs. In addition, Nestin is up-regulated in injured adult tissues, where its expression is thought to contribute to tissue regeneration. One example of Nestin up-regulation in adult tissue is the formation of glial scars following central nervous system injury [13]. These findings strongly suggest that Nestin has an important role in controlling cell proliferation.

Unlike terminally differentiated somatic cells which proliferate only under exposure to exogenous growth signals, stem cells are able to stimulate their own proliferation by expressing endogenous growth signals. Differential expression of Nestin in NPCs and mature cells makes the protein a good candidate for investigations on how to regulate proliferation of NPCs. Recent studies have indicated that Nestin plays an important role in organizing and modifying signaling pathways for cell proliferation, migration, and differentiation [14–17]. In rat neuronal cells, Cyclindependent kinase 5 (CDK5), an important cofactor of Nestin, mediates Nestin phosphorylation and activation to regulate cell survival under oxidative stress [16, 18]. A recent report has shown that in human A172 glioma cells, Nestin acts to keep cells in a highly proliferative state by sequestering the glucocorticoid receptor (GR) within the cell cytoplasm [19]. Retention of GR by Nestin prevents its translocation to the nucleus and thereby blocks GR-induced growth arrest [19]. Another study conducted in zebra fish has demonstrated that suppressed Nestin levels lead to developmental defects of the brain and eye during embryogenesis [20]. Despite the extensive studies indicating that Nestin is associated with proliferating cells, little is known about the molecular mechanism of Nestin in mammalian neural development. Interestingly, two recent Nestin knockout studies showed opposite results: one suggested that Nestin is required for the proper self-renewal of neural stem cells [21], and the other indicated that Nestin is not essential for development of the CNS [22]. Therefore, studies which focus on signaling pathways associated with Nestin in NPCs will greatly enhance our understanding of the behaviors of these cells at the molecular level.

In this study, we used mouse embryonic cortical cell-derived neurospheres as NPCs to investigate the function of Nestin in proliferating cells. We found that shRNAs directed against Nestin inhibited NPCs proliferation and cell cycle progression, reduced the expression of epidermal growth factor receptor (EGFR), and blocked EGF-induced proliferation and signaling pathways. Furthermore, we discovered that the p38-MAPK inhibitor PD169316 could rescue the growth arrest induced by shRNAs directed against Nestin. These findings suggest an important regulative pathway of Nestin-p38-EGFR in NPCs proliferation and neural development.

## RESULTS

### Nestin knockdown induces growth arrest of mouse NPCs *in vitro*

**Figure 1:**
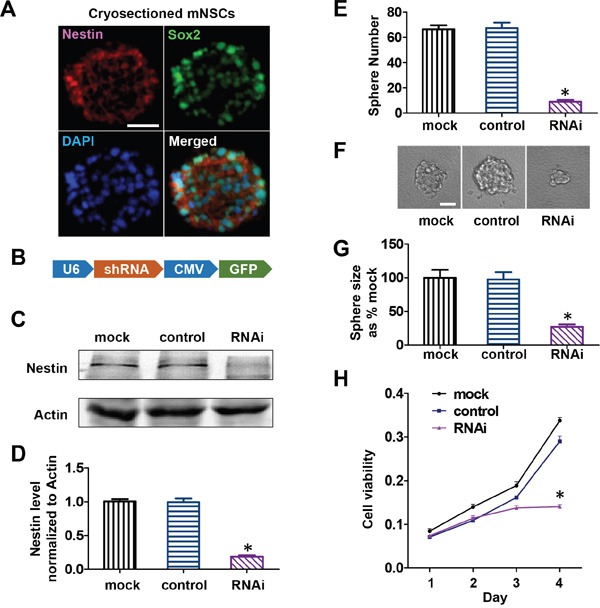
Nestin knockdown-induced growth arrest of mNPCs *in vitro* **A**. Double immunofluorescence staining showing that more than 70% of mNPCs showed stable expression of progenitor markersNestin and Sox2. Bar=20 μM. **B**. Adenoviral construct for Nestin shRNA expression. **C**. Immunoblots showing the knockdown efficiency of Nestin shRNA adenovirus in mNPCs. The scramble virus and uninfected cells were included as controls. **D**. Quantitative analysis of mNPCs showing an 80% reduction in Nestin expression after infection byNestinshRNA adenovirus for 72 hours. (*p<0.01, n=3) **E**. Sphere forming assay showing an 80% reduction in the number of mNPCs in the Nestin knockdown group compared with control groups. (*p<0.01, n=5) **F** and **G**. Sphere forming assay showing a 73% reduction in the size of neurospheres in the Nestinknockdown group compared withneurospheres in the control groups. Bar=20 μM. (*p<0.01, n=5) **H**. Cell viability was significantly reduced in mNPCs infected with NestinshRNA adenovirus for 72 hours compared to controls.(*p<0.01, n=3).

To investigate the function of Nestin in NPCs, we used freshly isolated mouse E13 cortical progenitors as mNPCs in this study. Double immunofluorescence staining revealed that ≥ 90% of cells in cultured neurospheres stained positive for Nestin and Sox2, two important markers of mNPCs (Figure 1A). Cells were successfully differentiated into neurons and astrocytes, confirming that the mNPCs were of neurogenic lineage. Upon differentiation, Nestin expression was down-regulated in these cells while the expression of other intermediate filaments such as neurofilament and glial fibrillary acidic protein were up-regulated (Supplementary Figure S1). Based on these findings, we hypothesized that Nestin expression was required to maintain mNPCs in the undifferentiated state and to retain their ability to self-renew and proliferate. To test this hypothesis, we generated Nestin-shRNA adenoviral vectors for loss of function investigation. mNPCs were infected with shRNA adenovirus targeted against Nestin. Seventy two hours post-infection, Nestin expression was reduced by ≥ 80% compared to control groups (transduced with adenoviral vectors expressing non-specific scramble shRNA), and the Mock (untreated cells) showed no significant difference (Figure 1C & 1D). To test the effects of Nestin knockdown on the ability of mNPCs to self-renew, we performed a sphere forming assay. E13 cortical cells infected with Nestin-shRNA were plated at a density of 1000 cells/well in a 96 well plate. Seven days post-infection, shRNA treatment reduced the number and size of neurospheres by 70% compared to uninfected control cells or cells infected by scramble virus (Figure 1E, & 1G). Furthermore, cell viability assays revealed that Nestin-depleted cells showed impaired cell proliferation 3 days after transduction compared to the non-infected and scramble control groups (Figure 1H).

### Nestin knockdown reduces mNPC engraftment *in vivo*

**Figure 2:**
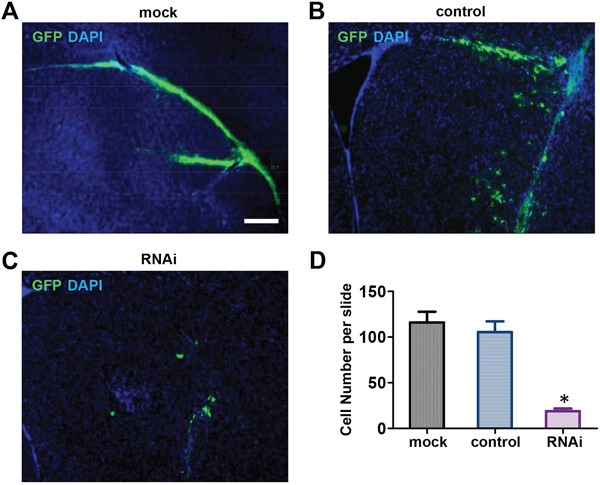
Nestin knockdown suppresses mNPCs Growth *in Vivo* **A, B, C**. Seven days post-injection, the Nestin knockdown group (C) showed negligible numbers of GFP-positive cells engrafted in brain tissue compared to the control (B) and Mock (A). Bar=200 μM. **D**. Quantitative analysis showing that the number of GFP-positive cells in the Nestin knockdown group was reduced by approximately 85% compared to the control.(*p<0.01, n=3).

**Figure 3:**
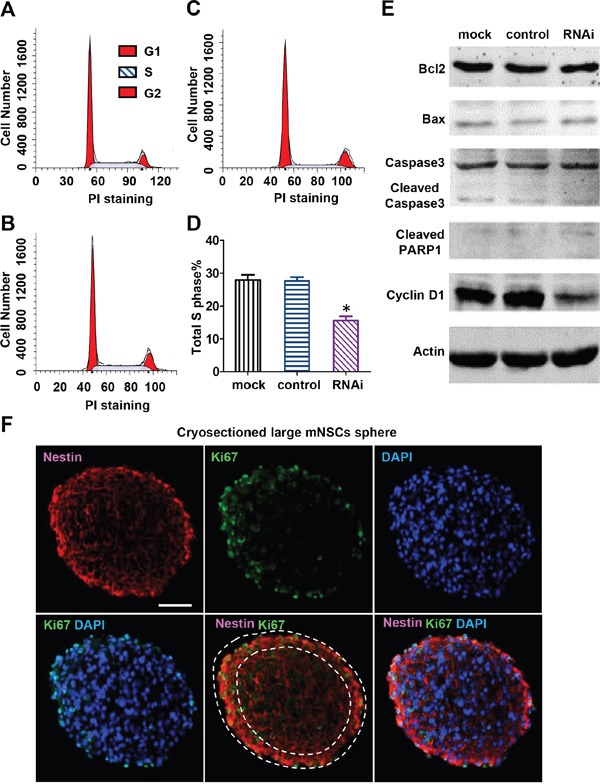
Nestin knockdown reduces cyclin D1 expression and induces cell cycle arrest of mNPCs Flow cytometry analysis of mNPCs in the mock group **A**. control group **B**. and Nestin knockdown group **C**. The percentage of G1, S and G2 cells were respectively 53.29, 28.62, and 12.09% in the mock group, 56.30, 27.71, and 13.49% in the control group, and 67.42, 15.71, and 16.86% in the Nestin knockdown group. Sub-G1 cells (apoptotic cells) were not detected in all three groups. **D**. S-phase analysis showing a 50% reduction in the number of S phase cells in the Nestin knockdown group compared with control. (*p<0.01, n=3) **E**. Western blot of cyclin D1 and apoptosis proteins: Seventy two hours post-infection, cyclin D1 expression level in the Nestin knockdown group was significantly reduced compared with control. No significant changes were observed in the expression levels of apoptosis-associated proteins Bcl-2, Bax, cleaved caspase 3, and cleaved PARP in Nestin knockdown mNPCs compared to mock and control. **F**. Double immunofluorescence staining showing colocalization of Nestin and proliferation marker Ki67 in the mock group uninfected with virus. Bar=20 μM.

Based on our *in vitro* data, we hypothesized that Nestin knockdown also suppresses NPC proliferation *in vivo*. To test this hypothesis, E13 cortical cells were isolated from GFP-positive transgenic mice and infected with shRNA adenovirus against Nestin for *in vivo* experimentation; control cells were infected with control adenovirus. GFP-positive mNPCs (10^5^ cells) were injected into the right striatium of 4 week old female nude mice (N=6 mice per group). Seven days post-injection, mice that received Nestin knockdown cells showed much fewer grafted GFP-positive cells (Figure 3C) compared to the mock and scramble control groups (Figure 2A, 2B & 2C). Quantitative analysis of images showed that the number of successfully grafted GFP-positive cells in the Nestin knockdown group was reduced by 85% compared to the control (Figure 2D). This data showed that Nestin knockdown strongly suppresses the proliferation of NPCs *in vivo*.

### Nestin knockdown induces cell cycle arrest in mNPCs

Cell cycle arrest and apoptosis are two common causes of cell viability reduction. To determine which pathway is associated with cell death observed in Nestin knockdown NPCs, we examined the cell cycle of Nestin-depleted mNPCs by flow cytometry (Figure 3A, 3B, 3C). The number of S phase cells in Nestin knockdown NPCs was significantly reduced (50% decrease) compared to control (Figure 3D). The expression of CyclinD1, a key cell cycle regulator, was examined in Nestin knockdown mNPCs by western blot. mNPCs infected with shRNAs against Nestin for 72 hrs showed significantly reduced expression of Cyclin D1 compared to the control groups (Figure 3E). This data demonstrated that Nestin knockdown suppresses NPC proliferation by reducing Cyclin D1 expression.

To determine whether Nestin knockdown induces apoptosis, the expression of apoptosis proteins in Nestin knockdown mNPCs was examined by western blot. Nestin knockdown did not activate apoptosis in mNPCs, as evidenced by no significant changes in the expression levels of apoptosis regulators Bcl-2 and Bax, and undetectable levels of caspase 3 and PARP (Figure 3E). Consistent with western blot results, no sub-G1 cells were detected in Nestin knockdown mNPCs, as confirmed by flow cytometry (Figure 3A, 3B, 3C). Moreover, double immunofluorescence staining for Nestin and Ki67 revealed that cells with stronger Nestin expression also had higher Ki67 expression (Figure 3F). These results indicated that growth arrest is the main cause of Nestin knockdown-induced impairment of cell viability.

### Nestin knockdown down-regulates EGFR expression in mNPCs

**Figure 4:**
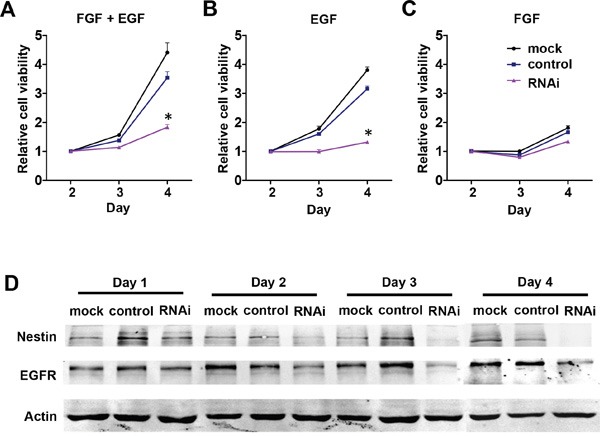
NestinNestin knockdown downregulates EGFR expression in mNPCs **A, B, C**. Cell viability of mNPCs under normal culture conditions with 20 ng/ml bFGF and EGF (left panel), in the presence of EGF only (center panel), and in the presence of bFGF only (right panel). Under normal culture conditions, the viability of mNPCs was significantly reduced in the Nestin knockdown group compared with control. In the presence of EGF only, the viability of mNPCs in both the Nestin knockdown and control groups was only slightly reduced as compared to normal culture conditions. In the presence of bFGF only, the viability of mNPCs in the Nestin knockdown and control groups was rapidly reduced and the pattern of growth curves was similar to the Nestin knockdown group under normal culture conditions. (*p<0.01, n=3) **D**. Western blot of EGFR indicates that expression of this receptor was significantly reduced overtime in the Nestin knockdown group compared with control.

Since basic fibroblast growth factor (bFGF) and epidermal growth factor (EGF) are two key factors that sustain NPC proliferation, we hypothesized that removal of either bFGF or EGF from the cells’ environment may affect Nestin knockdown-mediated growth arrest in NPCs. To test this hypothesis, we removed bFGF and EGF respectively from culture media and examined the effects on cell viability. Under normal culture conditions (with 20 ng/mL bFGF and EGF), cell viability was significantly reduced in the Nestin knockdown group compared to control and mock (Figure 4A). With removal of bFGF from culture medium, cell viability of all treatment groups followed similar trends as in the normal culture condition, indicating that bFGF signaling was not involved in the mechanism of Nestin knockdown-induced cell cycle arrest (Figure 4B). Interestingly, with removal of EGF from culture medium, cell viability of the Nestin knockdown and control groups were significantly reduced and the growth curves close resembled those of the Nestin knockdown group in normal culture conditions (Figure 4C). This data indicated that knockdown of Nestin or removal of EGF from culture medium induced similar patterns of cell growth arrest, which indicated that Nestin knockdown suppresses EGF-related proliferation and signal transduction in mNPCs. To further investigate the molecular mechanism, the expression of EGF receptor (EGFR) was analyzed by western blot. Results indicated that the expression of EGFR was significantly reduced in the Nestin knockdown group overtime compared with the control, which suggested that Nestin knockdown induces growth arrest of mNPCs by blocking the EGF-EGFR signaling pathway (Figure 4D).

### Inhibition of P38 activity rescues Nestin knockdown of NPC phenotype

**Figure 5:**
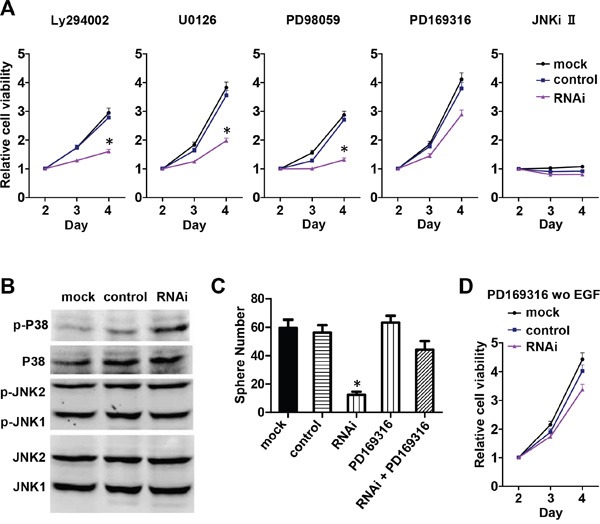
Nestin knockdown upregulates P38 activity and impairs mNPC self-renew and proliferation **A**. Viability of mNPCs treated with inhibitors of MAPK activity. Compared with mNPCs under normal culture conditions (Figure 5A), the viability of mNPCs in the Nestin knockdown and control groups was slightly reduced after treatment with PI3K inhibitor LY294002, MEK1/2 inhibitor U0126, and MEK1 specific inhibitor PD98059 respectively (left three panels). After treatment with JNK inhibitor (right most panel), the viability of mNPCs in both the Nestin knockdown and control groups was significantly reduced. After treatment with P38 inhibitor PD169316 (second right panel), the viability of mNPCs in the Nestin knockdown group was significantly increased and the growth curve showed a similar profile as control. **B**. Phosphorylated P38 expression was significantly increased in Nestin knockdown cells compared with control. mNPCs in the Nestin knockdown group showed similar levels of phosphorylated JNK (p-JNK/JNK 1/2) as the control. **C**. Sphere forming assay reveals that the P38 inhibitor PD169316 could significantly increase the viability of mNPCs in the Nestin knockdown group. (*p<0.01, n=5) **D**. In the absence of EGF, P38 inhibitor PD169316 could rescue the effect of Nestin knockdown on cell viability.

The mitogen-activated protein kinase (MAPK) and phosphoinositide-3 kinase/protein kinase B (PI3K/Akt) pathways are the two major downstream pathways of EGF signaling that control cell proliferation. To investigate which pathway is involved in Nestin knockdown-induced cell cycle arrest, mNPCs were treated with PI3K, MEK1/2, P38, and JNK inhibitors. Compared to untreated cells (Figure 5A), the proliferation rates of NPCs in the mock and scramble control groups were reduced when these cells were treated with PI3K inhibitor LY294002 (10 μM), MEK1/2 inhibitor U0126 (1 μM), and MEK1-specific inhibitor PD98059 (10 μM) (Figure 5A). When treated with JNK inhibitor (10 μM), all groups showed complete growth arrest, indicating an indispensable role that JNK plays in the proliferation of NPCs which is not associated with Nestin knockdown (Figure 5A). Most importantly, when treated with P38 inhibitor PD169316 (10 μM), Nestin knockdown cells showed a significantly increased rate of proliferation which approached the growth curve of control cells (Figure 5A); this indicates that the P38 inhibitor reversed the effect of Nestin knockdown. To further investigate the activity of P38 and JNK in Nestin-depleted cells, phosphorylation of these two kinases was examined by western blot. Consistent with previous results, phosphorylated P38 expression was significantly increased in Nestin knockdown cells compared to both control groups (Figure 5B). However, JNK1/2 showed the same level of phosphorylation in both the control groups and the Nestin knockdown group. In addition, our sphere forming assay revealed that the P38 inhibitor restored the number of neurospheres in the Nestin knockdown group to the same level as the controls (Figure 5C). Meanwhile, P38 inhibitor PD169316 could rescue the effect of Nestin knockdown on cell viability in the absence of EGF. These results indicate that P38 signaling acts as a negative regulator of NPC proliferation, and that JNK activation is required to maintain NPC proliferation (Figure 5D).

### Nestin knockdown promote neuronal differentiation of NPCs

**Figure 6:**
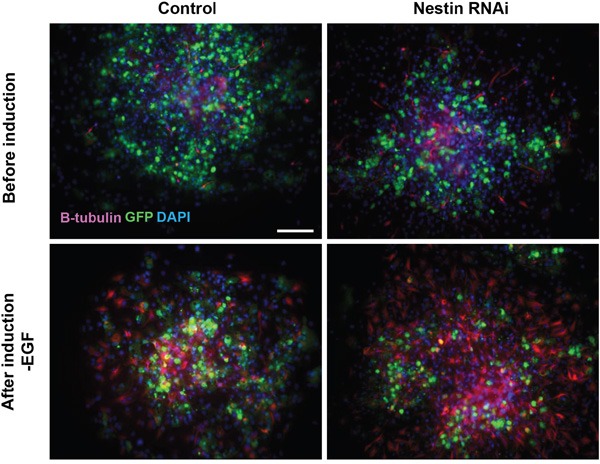
Nestin knockdown promote neuronal differentiation of NPCs Immunofluorescence staining showing that more beta-tubulin III positive neurons (red) derived from NPCs treated with Nestin shRNA. Before induction upon withdrawal of EGF, there were neurons in Nestin knockdown NPCs attached on matrigel, and much more after induction than the control. Bar=100 μM.

Beside the impairment of cell viability, it is important to understand the impact of Nestin knockdown on the stemness of NPCs especially differentiation. To investigate whether Nestin knockdown affect the ability of NPCs in differentiation, we performed spontaneous differentiation of on NPCs. Before induction upon withdrawal of EGF, we already observed many beta-tubulin III positive neurons in Nestin knockdown NPCs when attached on matrigel. Moreover, we found much more neurons were after induction than the control (Figure 6). These results suggest that Nestin knockdown promote neuronal differentiation of NPCs.

## DISCUSSION

This study has revealed that Nestin plays a critical role in maintaining the self-renewal and proliferation of NPCs via the P38-EGFR signaling pathway. Knockdown of Nestin expression by shRNA results in growth arrest of mNPCs, which is mediated by increases in p38 MAP activity and reductions in EGFR expression. Both *in vitro* and *in vivo* data strongly indicate that Nestin is an essential factor that regulates NPC proliferation. Using the same mouse strain and similar *in vitro* NPCs model, our results are consistent with the knockout study by Park et al [21]. Meanwhile, contradictory *in vivo* results presented in the other knockout study by Mohseni et al [22], which did not look into the behaviors of cultured NPCs, could be caused by different methods of generating knockout mouse.

Nestin regulates proliferation, migration, and apoptosis in various cell types. It protects NPCs from oxidation-induced apoptosis by suppressing Cdk5 activity [18]. EGF-induced expression of Nestin in rat vascular smooth muscle cells protects these cells against apoptosis by inhibiting Cdk5 activity, which leads to up-regulation of the anti-apoptotic protein Bcl-2 [23]. The anti-apoptotic role may also contribute to the Nestin knockdown engraftment reduction shown in our *in vivo* data. Nestin also plays a critical role in regulating cell motility and proliferation [24]. For instance, prostate cancer cells lose their ability to migrate after treatment with siRNA against Nestin [11]. Human A-172 glioma cells undergo growth arrest after suppression of Nestin [19], and astrocytoma cells cease to grow after treatment with siRNA against Nestin [25]. Nestin suppression attenuates invasive potential of endometrial cancer cells by downregulating TGF-β signaling pathway [26]. Nestin also promotes the proliferation of mesangial cells [27]. These studies collectively indicate that Nestin plays an important role in NPC proliferation, growth, and survival. However, the mechanism of how Nestin regulates the proliferation of NPCs is unclear. Our study indicates that Nestin expression is highly correlated with the proliferative activity of NPCs as indicated by co-localized expression of Nestin and Ki67 (Figure 3F). In this study, we have demonstrated that shRNA against Nestin suppresses proliferation and self-renewal of mNPCs and that the expression of Nestin in NPCs is crucial to maintaining their progenitor/stem cell properties.

Epidermal growth factor (EGF) is necessary for driving the proliferation and self-renewal of NPCs [28–30]. Neonatal mice lacking EGFR undergo rapid neurodegeneration during the first four days after birth due to massive apoptosis of neural cells [31]. Our unpublished data also implicate that FGF is required for high passage NPCs after prolonged culture, while EGF is more important in stimulating the expansion of freshly isolated NPCs. EGFR and Sox2 form a feedback loop that positively regulates NPC proliferation and self-renewal [32]. Activation of EGFRvIII expression enhances NPC proliferation and survival [33]. These studies suggest that EGFR is tightly associated with NPC proliferation. However, the mechanism by which EGFR regulates proliferation of NPCs is unclear. Our study identifies the relationship between Nestin and EGFR in NPCs. The results demonstrate that Nestin is essential to maintain EGFR expression in mNPCs, which in turn is required for NPCs to proliferate and self-renew.

P38 is a stress response protein which is activated by environmental stress signals and proinflammatory cytokines [34]. It is also associated with proliferation and differentiation in several types of cells [35, 36]. In the central nervous system, P38 is expressed in neurons and oligodendrocytes in the brain and is associated with cell survival [37–39]. Mice lacking p38 develop massive clusters of apoptotic cells in the neural tube and developmental defects of the placenta [40], vascular system [41], and myocardium [42, 43]. These findings suggest that P38 plays a major role in development. Some studies have reported that P38 negatively regulates the proliferation of NPCs during early brain development [44]. Moreover, P38 regulates EGFR degradation and has a profound impact on the cellular outcome of EGFR signaling [45]. In addition, we have found that c-Jun N-terminal kinases (JNKs) are necessary factors for mNPC proliferation. Studies have shown that JNK1 and JNK2 knockout mice exhibit embryonic lethality due to severe dysregulation of apoptosis in the brain [46] and neural tube closure, which resulted in fatally impaired neurodevelopment [47]. Another study has shown that JNK1 deletion severely inhibited neurogenesis of embryonic stem cells [48]. Consistent with these studies, our results have shown that JNKs are highly phosphorylated in NPCs, and that inhibition of JNKs could prevent mNPC proliferation. These results indicate that JNKs are indispensable regulators of mNPC proliferation in neural development, and that knockout of JNKs results in deficient NPC proliferation.

In conclusion, we have demonstrated that shRNA against Nestin downregulates EGFR expression in NPCs by activating P38, and that the Nestin-P38-EGFR pathway plays an important role in regulating the proliferation and self-renewal of NPCs during neural development. We have also shown that JNK signaling is an essential factor in controlling normal proliferation of NPCs. With a better understanding of the mechanisms regulating endogenous adult NPC functions, we can improve insights into the possible applications of stem cell therapies for neural diseases.

## MATERIALS AND METHODS

### Neural progenitor cell culture

Mouse neural progenitor cells (mNPCs) were isolated from the cortex of 3-day old neonatal mice as described previously [49]. Cortices were removed and dissociated with a fire-polished glass pipette, passed through a 40 μm mesh filter and resuspended at 50,000 cells/ml in DMEM/Ham's F12 medium (Invitrogen, Carlsbad, CA) containing B27 (Invitrogen), penicillin/streptomycin (Invitrogen), 5 μg/ml heparin (Sigma, St. Louis, MO), 20 ng/ml bFGF (Peprotech, Rocky Hill, NJ), and/or 20 ng/ml EGF (Peprotech, Rocky Hill, NJ). After 7 days in culture, neurospheres were dissociated into single cells using Accutase (Sigma, St. Louis, MO) and resuspended in the same medium at 50,000 cells/ml. For differentiation, clonal neurospheres in DMEM/Ham's F12 with B27, penicillin/streptomycin, 40 μM L-glutamine, and 25 mM glutamic acid (Sigma) were plated on poly-L-lysine (Sigma,)-coated (10 μg/ml) coverslips for 5 days. Immunohistochemistry was performed with antibodies (Chemicon, Temecula, CA) directed against β-tubulin III (TuJ1) and glial fibrillary acidic protein (GFAP) to identify neurons and astrocytes respectively. Human fetal cortical neural progenitor cells were prepared as previously described [50]. For sphere forming assay, hNPCs of passage 2-4 were dissociated into single cells in suspension and seeded at 1000 cells/well in 96 well plate. All conditions were done in duplicate and repeated 2–4 times. Human embryonic cortical tissues were dissected from normal aborted fetuses at 16 weeks of gestation after maternal consent and with the approval of the Peking University Health Science Center's Ethical Committee. Striatal progenitor cells (neurospheres) were obtained and cultured following the procedure described by Svendsen et al [51].

### Xenografts

NPCs isolated from GFP trangenic mice were used for transplantation after 3-4 passages (3-4 weeks *in vitro*). Cells were prepared for transplantation by first digesting the growing spheres to a single cell suspension. After assessment for viability, the cells were resuspended in DMEM to give a concentration of 75,000-125,000 cells/μl. Two microliters of the final suspension were then transplanted into the striatum of 6 week old nude mice (Animal Center of Peking University Health Science Center, Beijing, China).

### Vector production and viral packaging

The short hairpin RNA expression vector pDC316-GFP-U6 was constructed by inserting a GFP-coding sequence and anti-shRNA-expressing cassette with U6 promoter into the adenoviral shuttle plasmid pDC316 (Microbix, Toronto, Canada). A self annealing oligonucleotide targeting mouse Nestin (target sequence: AAG ATG TCC CTT AGT CTG GAG) or human Nestin (target sequence: AAG ATG TCC CTT AGT CTG GAG) was inserted into the vector pDC316-EGFP-U6. Scramble oligonucleotide having no homology with human, mice, or rat species was used to generate a control vector. Recombinant adenovirus was generated by co-transfection of backbone plasmid pBHGloxΔE1 3Cre and pDC316-EGFP-U6. Adenovirus was propagated in HEK 293 cells and purified with the AdenopureTM adenovirus purification kit (Puresyn, Malvern, PA). Viral titers were measured by serial dilution on NIH 3T3 cells followed by flow cytometric analysis after 48 hr. For viral transduction, adenoviral vectors at a multiplicity of infection (MOI) of 10 were added to dissociated NPCs prior to or just after plating.

### Western blots

Cells were lysed in 0.1% SDS solution with protease inhibitor cocktail (Roche, Indianapolis, IN) and the lysates analyzed by immunoblotting (30μg/lane). Primary antibodies used included anti-Nestin, anti-Bax, anti-Bcl2, anti-CyclinD1, anti-Caspase3, anti-Cleaved-PARP, anti-EGFR, anti-Actin (Santa Cruz Biotechnology, Santa Cruz, CA), anti-P38, anti-phospho-P38, anti-JNK, and anti-phospho-JNK (Cell signaling, Danvers, MA). Secondary antibodies used were IRDye 800 anti-mouse and IRDye 800 anti-rabbit molecular probes (Rockland Immunochemicals Inc, Limerick, PA). The Odyssey infrared imaging system (LI-COR, Biosciences, NE) was used to visualize immuno-reactive proteins on nitrocellulose membrane, and the acquired images were analyzed using the Odyssey software program according to software instructions.

### Cell viability assay

Cell proliferation assays were performed using the Cell Counting Kit-8 (Dojindo, Kumamoto, Japan). Single dissociated mNPCs were seeded (2000 cells/100μl) in a 96-well plate. For hNPCs, neurospheres were sectioned into 100 μm-thin sections that were seeded at a density equivalent to 5000 cells/100μl) in a 96-well plate [51]. Cell proliferation assays were performed using the CCK-8 kit according to the manufacturer's instructions. All conditions were done in duplicate and repeated 3 times.

### Immunofluorescence staining

Cryosectioned neurospheres and differentiated cells were fixed with 4% PFA and incubated with primary antibodies mouse anti-mouse Nestin (Santa Cruz Biotechnology), mouse anti human Nestin (Chemicon), rabbit anti-Sox2 (Chemicon), or mouse anti-Ki67 (Santa Cruz Biotechnology), and subsequently with secondary antibodies tetramethylrhodamineisothiocyanate-labeled goat anti-rabbit IgG or fluorescein isothiocyanate-labeled goat anti-mouse IgG (Santa Cruz Biotechnology). Photographs were taken on Olympus KX85 immunofluorescent microscope and Apogee Instruments Microscopy Fluorescence System (Model KX85, Japan).

### Cell cycle analysis

48 hours after adenoviral transduction, NPCs were collected and dissociated into single cells in suspension. Cells were fixed with 70% ethanol and treated with RNase (Invitrogen) for 20 minutes before addition of 5μg/mL propidium iodide and analyzed by flow cytometry.

### Statistical analysis

Data are shown as means ± SEM of three independent experiments. Statistical analysis was performed with Student's paired t-test (two-tail) and ANOVA using Prism 5 (GraphPad).

## SUPPLEMENTARY FIGURE


