# Supplementary material for: Suppression of Nestin reveals a critical role for p38-EGFR pathway in neural progenitor cell proliferation
Source: Oncotarget. 2016 Nov 22;7(52):87052–63. doi: 10.18632/oncotarget.13498 (PMC5349970; doi:10.18632/oncotarget.13498)
Supplement: Supplementary file 1 [file oncotarget-07-87052-s001.pdf]

Suppression of Nestin reveals a critical role for p38-EGFR pathway in neural progenitor cell proliferation

SUPPLEMENTARY FIGURE

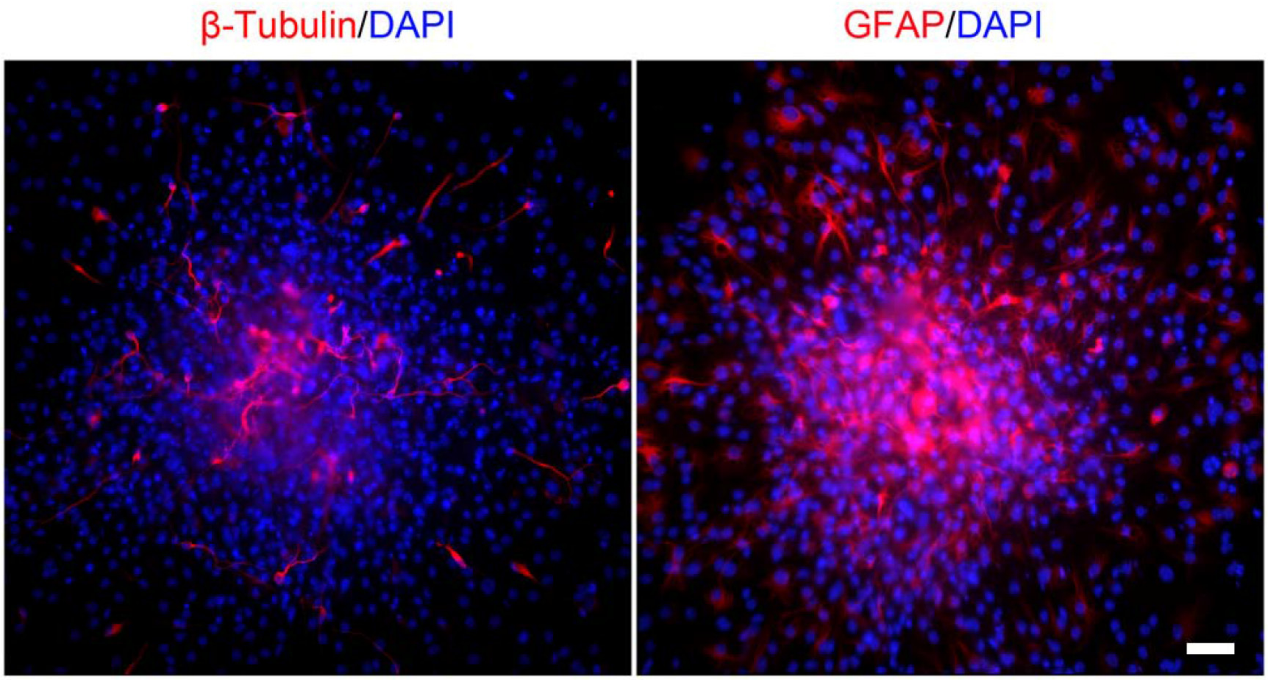

Supplementary Figure S1: Immunofluorescence staining showing NPCs can differentiate into beta-tubulin III positive neurons and GFAP positive astrocytes. Bar=20 μM.
